# Supplementary material for: Single Cell Transcriptomic and Chromatin Profiles Suggest Layer Vb Is the Only Layer With Shared Excitatory Cell Types in the Medial and Lateral Entorhinal Cortex
Source: Front Neural Circuits. 2022 Jan 26;15:806154. doi: 10.3389/fncir.2021.806154 (PMC8826650; doi:10.3389/fncir.2021.806154)
Supplement: Supplementary file 14 [file Data_Sheet_1.ZIP › Sup_vs_Deep_Motifs/homerResults/motif27.similar.html]

motif27

## Information for motif27

A
G
T
C
A
G
C
T
A
G
T
C
C
G
A
T
A
G
T
C
G
A
C
T
A
G
T
C
A
G
C
T
A
T
G
C
A
G
C
T
A
T
G
C
A
G
C
T
  
Reverse Opposite:  

C
T
G
A
A
T
C
G
C
T
G
A
A
T
C
G
C
T
G
A
T
C
A
G
C
T
G
A
T
C
A
G
C
G
T
A
A
C
T
G
C
T
G
A
A
C
T
G
  

|  |  |
| --- | --- |
| p-value: | 1e-7 |
| log p-value: | -1.658e+01 |
| Information Content per bp: | 1.811 |
| Number of Target Sequences with motif | 1352.0 |
| Percentage of Target Sequences with motif | 4.03% |
| Number of Background Sequences with motif | 298.4 |
| Percentage of Background Sequences with motif | 3.50% |
| Average Position of motif in Targets | 422.0 +/- 259.5bp |
| Average Position of motif in Background | 450.2 +/- 291.7bp |
| Strand Bias (log2 ratio + to - strand density) | 0.5 |
| Multiplicity (# of sites on avg that occur together) | 1.52 |
| Motif File: | file (matrix) reverse opposite |

### Similar de novo motifs found

|  |  |  |  |  |  |  |  |
| --- | --- | --- | --- | --- | --- | --- | --- |
| Rank | Match Score | Redundant Motif | P-value | log P-value | % of Targets | % of Background | Motif file |
| 1 | 0.863 | A G C T A G T C A C G T A T G C A C G T G T A C C A G T A T G C A C G T A G T C | 1e-6 | -15.203444 | 4.55% | 4.00% | motif file (matrix) |
| 2 | 0.746 | A C G T A G T C A C G T A G T C A C G T A G T C A C G T A G T C | 1e-1 | -2.902992 | 9.42% | 9.17% | motif file (matrix) |
